# On the Physiological Effects of Tobacco

**Published:** 1865-01

**Authors:** 


On the Physiological Effects of Tobacco.—*By Dr. Richardson.*—This paper is a very valuable addition to the literature of smoking, dealing with the composition of the products of combustion of tobacco, the physiological action of the various compounds thus derived, and the effects of ordinary and excessive smoking on the organs of the body. The following bodies are products of the combustion of tobacco: 1, water; 2, free carbon; 3, ammonia; 4, carbonic acid; 5, an alkaloidal principle called nicotine; 6, an empyreumatic substance; 7, a resinous bitter extract. The water is in the form of vapor; the carbon is in the form of minute particles, suspended through the water vapor, and giving to the eddies of smoke their blue color; the ammonia is in the form of gas combined with carbonic acid; the carbonic-acid gas is partly free and partly in combination with ammonia. The nicotine is a non-volatile body, an alkaloid which remains in the pipe; the empyreumatic substance is a volatile body, having the nature of ammonia, but the exact composition of which is as yet unknown. It is this that gives to the smoke its peculiar odor; it adheres very powerfully to woolen materials, and, in the concentrated form, is so obnoxious as almost to be intolerable. The bitter extract is a resinous substance, of dark color, and of intensely bitter taste. It is, probably, a compound body, having an alkaloid as its base. It is not volatile, and only leaves the pipe by being carried along the stem in the fluid form. The greatest variation exist in various kinds of tobacco. Simple tobacco that has not undergone fermentation yields very little free carbon, much ammonia, much carbonic acid, little water, none or the smallest possible trace of nicotine, a very small quantity of empyreumatic vapor, and an equally small quantity of bitter extract. Latakia tobacco yields these same products only. Bristol bird's-eye yields large quantities of ammonia, and very little nicotine. Turkish yields much ammonia. Shag tobacco yields all the products in abundance, and the same may be said of pure Havana cigars. Cavendish varies considerably; some specimens which are quickly dried are nearly as simple as Latakia; other specimens which are moist yield all the products in great abundance. Pigtail yields every product most abundantly. The little Swiss cigars yield enormous quantities of ammonia, and Manillas yield very little.

The physiological effects of these compounds are as follows: The water vapor is innocuous; the carbon settles on the mucuous membrane, and irritates the throat. The carbonic acid is a narcotic, if it be received into the lungs. The ammonia causes dryness and biting of the mucuous membrane of the throat, and increases the flow of saliva; absorbed into the blood, it renders that fluid too thin, causing irregularity of the blood corpuscles; it also causes, when absorbed in large quantities, suppression of the billiary secretion and yellowness of the skin; it quickens and then reduces the action of the heart, and, in young smokers, it produces nausea. The empyreumatic seems to be almost negative in its effects, but it gives to the tobacco-smoke its peculiar taste; and it is this substance that makes the breath of confirmed smokers so unpleasant. Nicotine is scarcely every imbibed by the cleanly smoker; it affects those only who smoke cigars by holding the cigar in the mouth, and those who smoke dirty pipes saturated with oily matter. Its effects when absorbed are very injurious; it causes palpitation, tremor, and irregular action of the heart; tremor and unsteadiness of the muscles generally, and great prostration. It does not, however, produce nausea or vomiting. The bitter extract is the cause of vomiting and nausea when it is absorbed; both it and the nicotine are always received into the mouth in solution, and produce their effects, either by direct absorption from the mouth, or by being imperceptibly swallowed and taken into the stomach.

The greatest difference arises from the manner of smoking. Those who use clean, long pipes of clay feel only the effects of the gaseous bodies and the free carbon. Wooden pipes and pipes with glass stems are injurious. Cigars smoked to the end are most injurious of all. To be safe, a cigar ought to be cast aside as soon as it is half smoked; and every cigar should be smoked from a porous tube. Cigars, indeed, are more injurious than any form of pipe; and the best pipe is unquestionably what is commonly called a "churchwarden," or "long clay." After the clay pipe, the meerschaum is the next wholesome. A pipe with a meerschaum bowl, an amber mouth-piece, and a clay stem, easily removable or changeable for a halfpenny, would be the beau ideal of a healthy pipe. A man may, by practice, become habituated to a short foul pipe, but he never fails to suffer from his success in the end, nor, unless the habit of actual stupefaction be acquired, is any pleasurable advantage derived.

The effects of moderate and immoderate smoking on the organs of the body were stated to be as follows: In an adult man, who is tolerant of tobacco, moderate smoking—say to the extent of three clean pipes of the milder forms of pure tobacco in the twenty-four hours—does no great harm. It somewhat stops waste, and soothes; but there are times when it unsettles the digestion. To an immoderate degree—say to six or eight pipes a day, especially if strong tobacco and fine pipes be used—smoking is unquestionably very injurious to the animal functions. The blood is made too fluid; the biliary secretion is arrested; and the digestion is constantly deranged; there is dryness of the tongue and frequent nausea. On the heart the symptoms are very marked. They consist of palpitation, a sensation as though the heart were rising upward, a feeling of breathlessness, and, in bad cases, of severe pain through the chest, extending through the upper limbs. The action of the heart is intermittent, and faintness may be experienced. Extreme smoking is also very injurious to the organs of sense. In all inveterate, constant smokers, the pupils of the eyes are dilated, owing to the absorption of nicotine, and the vision is impaired in strong light; but the symptom which most affects the vision is the retention of images on the retina after the eye is withdrawn from them. Thus, if he turn his eyes from a window, he retains the impression of the window, the panes seeming red and the bars dark. When such pictures are seen for some minutes, the smoker may be assured that he has carried his indulgence out of the pale of safety. On the sense of hearing inveterate smoking produces disturbances; these consist of restless deafness, and ringing or whistling in the ears. The circulation of the brain is also sometimes disturbed, and giddiness and vertigo are produced. The muscles, after extreme smoking, are prostrated. Long smoking also affects the mucous membrane of the mouth, causing "smoker's sore throat." There are also other effects occasionally produced in the mouth—viz., sponginess of the gums and tartar on the teeth. On the whole, however, smoking does not injure the teeth. These are the worst effects of tobacco; they all point to funtional disturbance. The question remains whether worse effects ever follow from over-dulgence in smoking. The great effect of tobacco is to arrest the functional processesses on which growth and development depend. To the whole body of the growing youth, therefore, the act of smoking is decidedly deleterious. Dr. Richardson could not dwell too forcibly on this point. He did not say that any one particular disease was brought on, but that a general deficiency of power was induced. When, however, the body has ceased to grow, the effects of tobacco in this regard were not felt; and, when the body was falling into decay, smoking seems conservative in its action. The author had met many instances of extremest old age in confirmed smokers. As regards the production of specific diseases by tobacco, the hypotheses that have been raised are too loose to be accepted. It is said that tobacco dulls and destroys the mental faculties. The facts are that, when the body is in full vigor, smoking does lessen the power of the faculties; but, when the body is overworked and worn, tabacco soothes and conserves. If there were any foundation in the idea that tobacco produces insanity, the fact would be at once broadly marked in the difference of numbers of the insane in the different sexes. This remark is, however, less applicable to paralysis. It has been urged that tobacco produces cancer; the statement is utterly groundless. Neither consumption nor bronchitis, in the chronic form, can be induced primarily by smoking. At the same time it must be added that smoking does mischief in both these disorders when they exist, except in asthma. In the main, smoking is a luxury which any man is better without. Of nearly every luxury, tobacco is the least injurious. It is innocuous as compared with alcohol; it does infinitely less harm than opium; it is in no sense worse than tea, and, by the side of high living, altogether contrast most favorably. *Proceedings of the British Association, in London Reader.*

